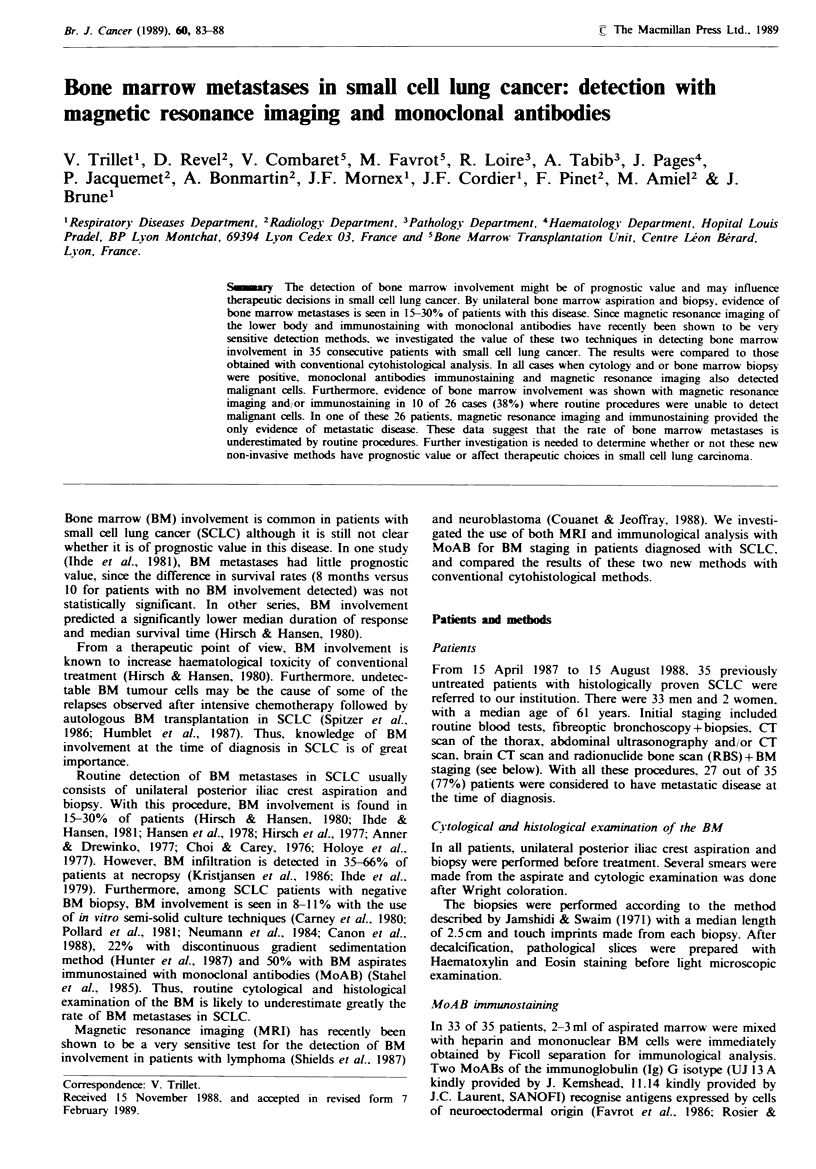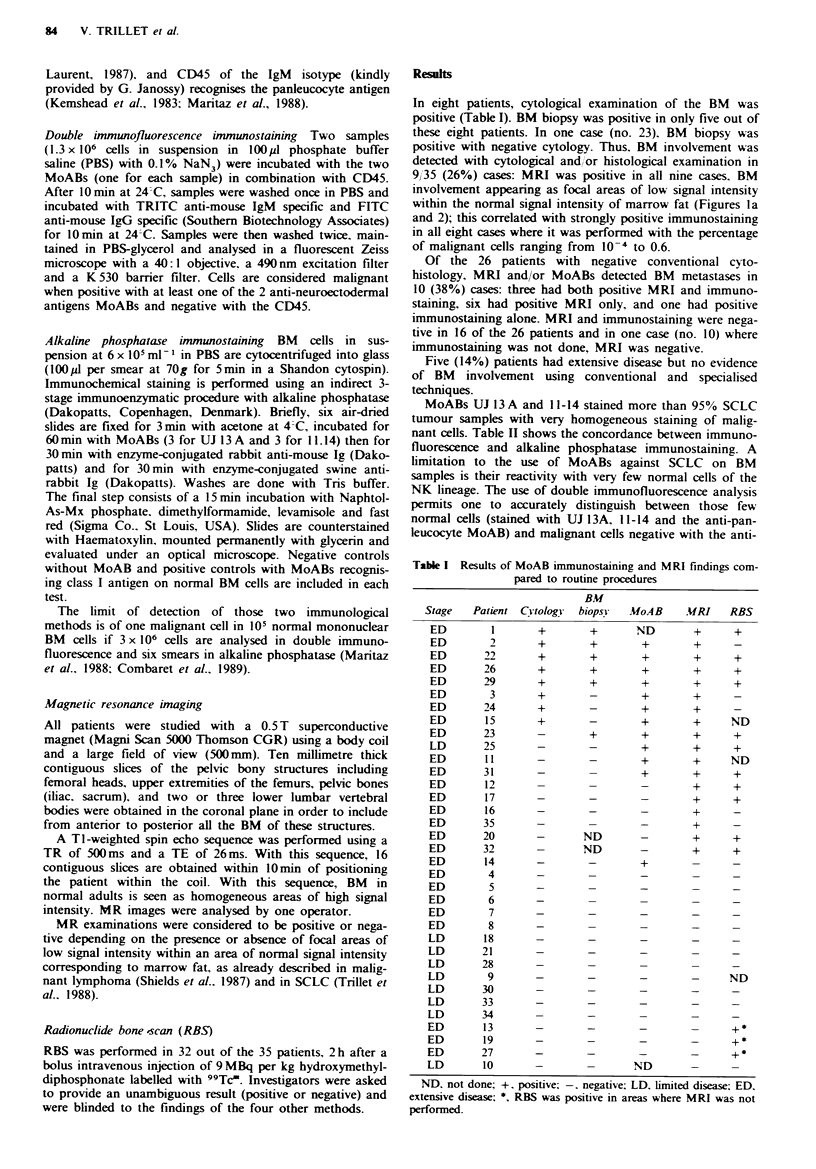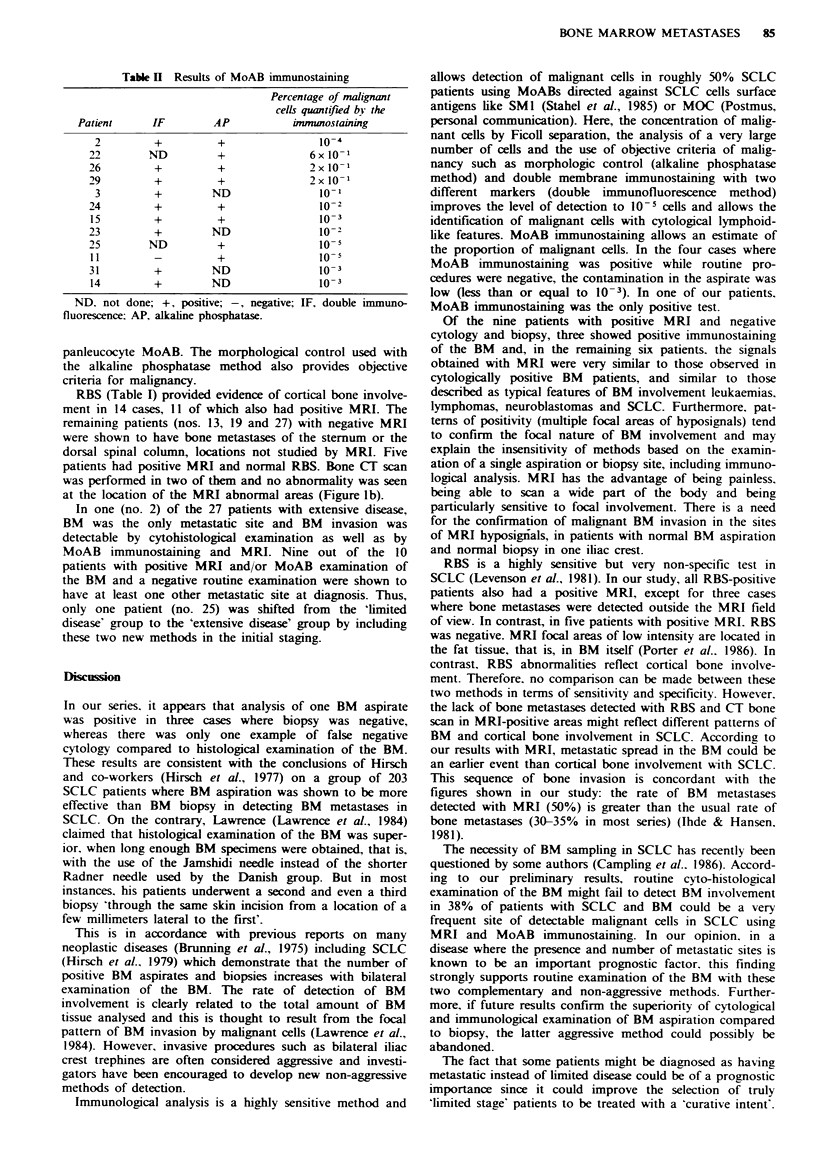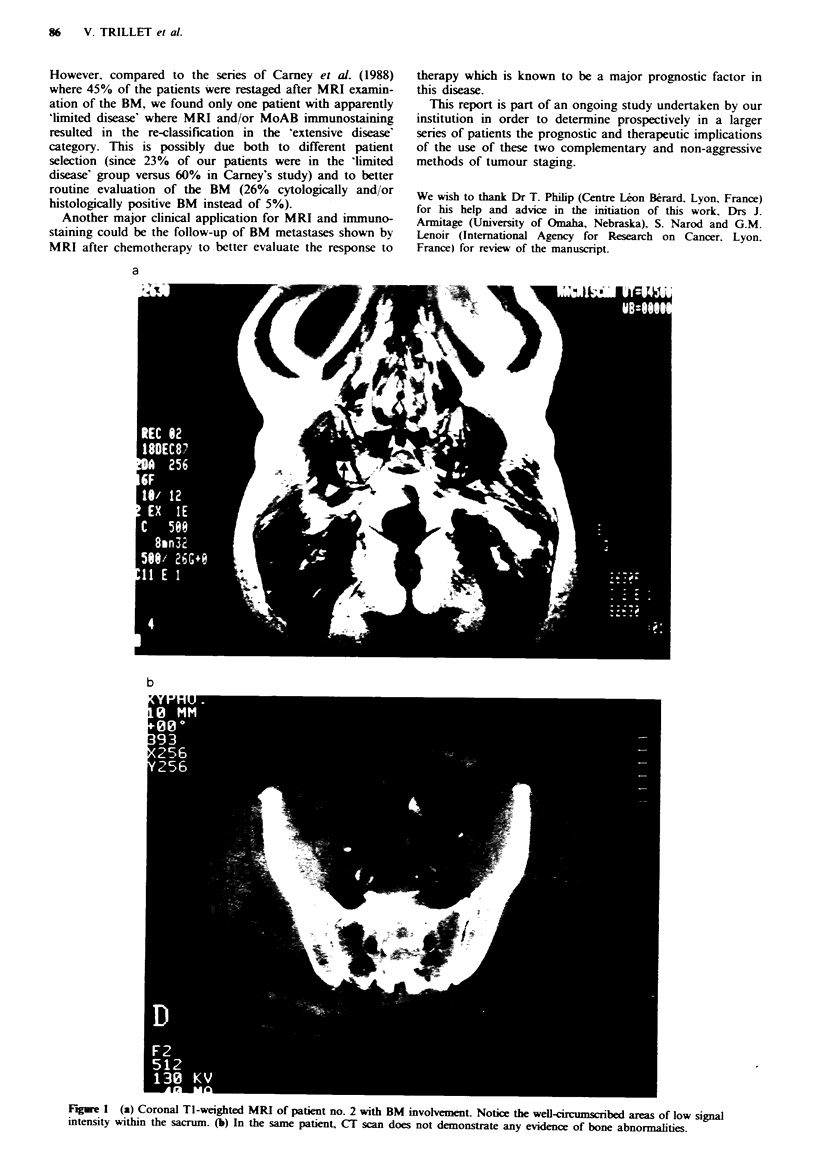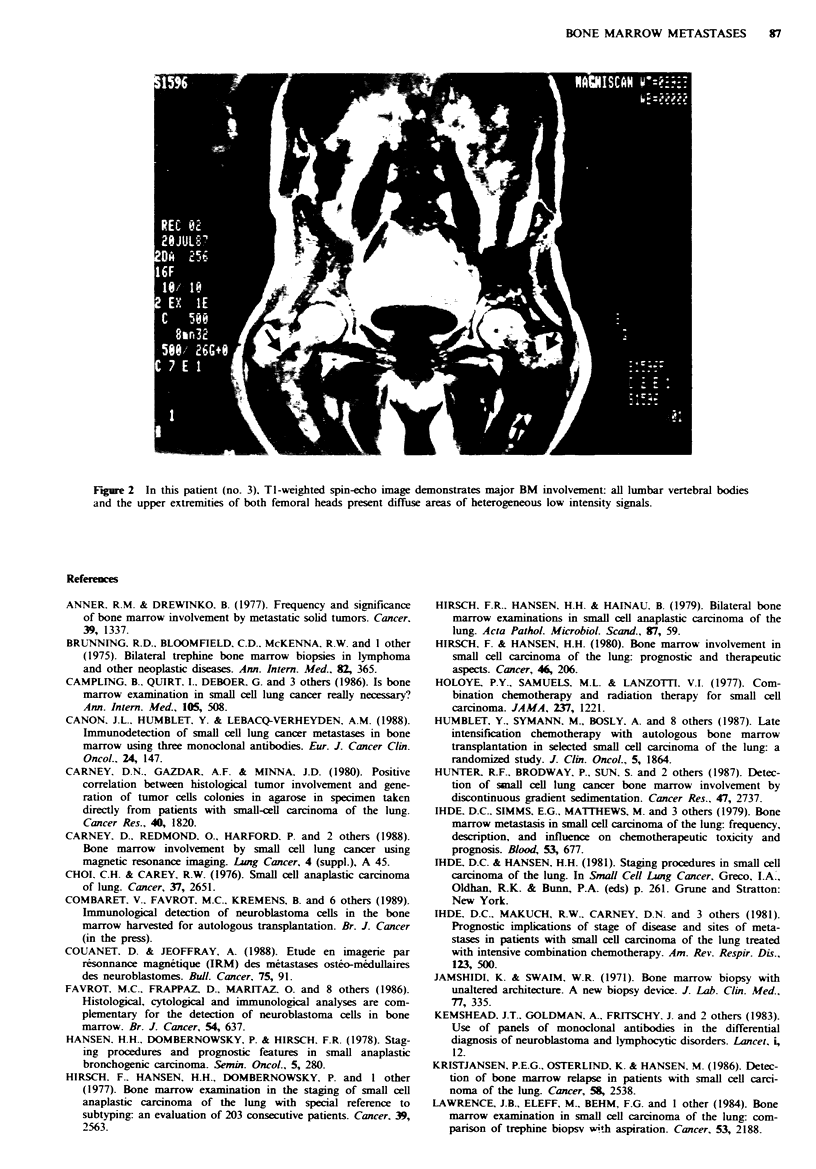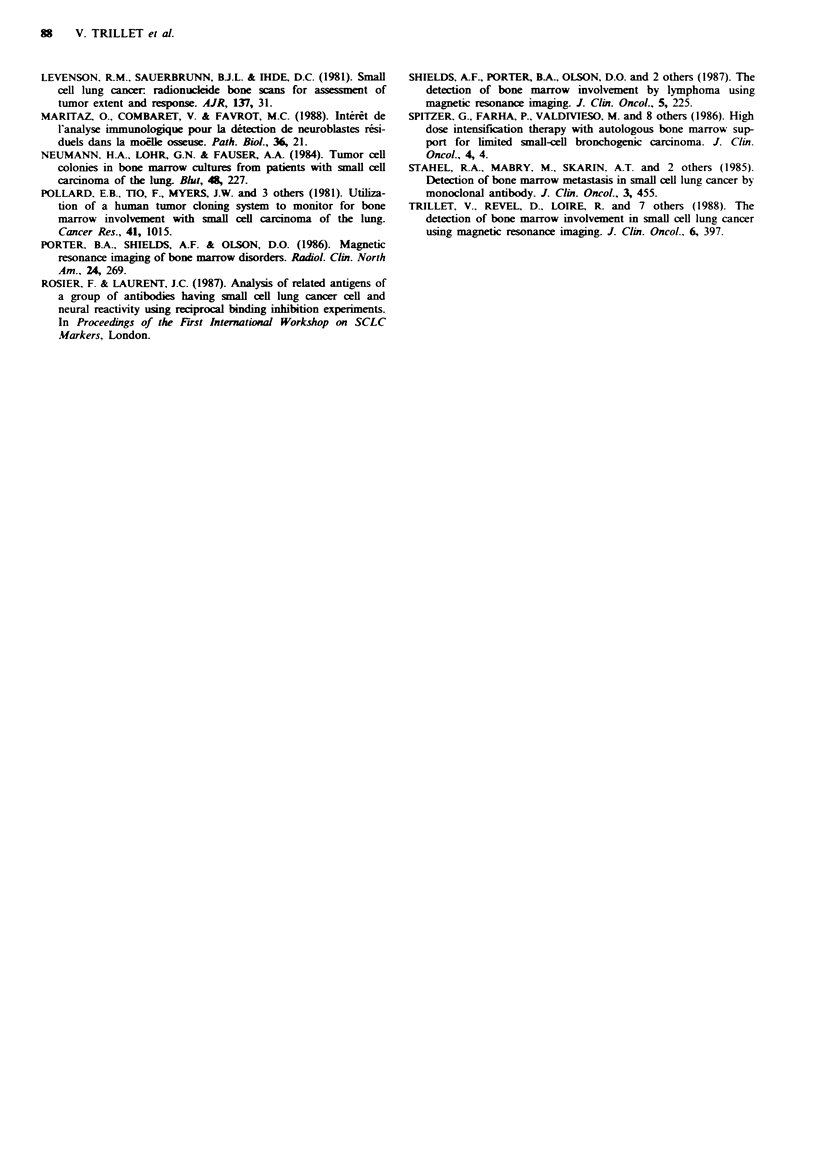# Bone marrow metastases in small cell lung cancer: detection with magnetic resonance imaging and monoclonal antibodies.

**DOI:** 10.1038/bjc.1989.225

**Published:** 1989-07

**Authors:** V. Trillet, D. Revel, V. Combaret, M. Favrot, R. Loire, A. Tabib, J. Pages, P. Jacquemet, A. Bonmartin, J. F. Mornex

**Affiliations:** Respiratory Diseases Department, Hopital Louis Pradel, Lyon, France.

## Abstract

**Images:**


Br. J. Cancer (1989), 60, 83-88 © The Macmillan Press Ltd., 1989

### Bone marrow metastases in small cell lung cancer: detection with magnetic resonance imaging and monoclonal antibodies

V. Trillet[1], D. Revel[2], V. Combaret[5], M. Favrot[5], R. Loire[3], A. Tabib[3], J. Pages[4], P. Jacquemet[2], A. Bonmartin[2], J.F. Mornex[1], J.F. Cordier[1], F. Pinet[2], M. Amiel[2] & J. Brune[1]

[1]*Respiratory Diseases Department*, [2]*Radiology Department*, [3]*Pathology Department*, [4]*Haematology Department, Hopital Louis Pradel, BP Lyon Montchat, 69394 Lyon Cedex 03, France and* [5]*Bone Marrow Transplantation Unit, Centre Léon Bérard, Lyon, France.*

**Summary** The detection of bone marrow involvement might be of prognostic value and may influence therapeutic decisions in small cell lung cancer. By unilateral bone marrow aspiration and biopsy, evidence of bone marrow metastases is seen in 15–30% of patients with this disease. Since magnetic resonance imaging of the lower body and immunostaining with monoclonal antibodies have recently been shown to be very sensitive detection methods, we investigated the value of these two techniques in detecting bone marrow involvement in 35 consecutive patients with small cell lung cancer. The results were compared to those obtained with conventional cytohistological analysis. In all cases when cytology and/or bone marrow biopsy were positive, monoclonal antibodies immunostaining and magnetic resonance imaging also detected malignant cells. Furthermore, evidence of bone marrow involvement was shown with magnetic resonance imaging and/or immunostaining in 10 of 26 cases (38%) where routine procedures were unable to detect malignant cells. In one of these 26 patients, magnetic resonance imaging and immunostaining provided the only evidence of metastatic disease. These data suggest that the rate of bone marrow metastases is underestimated by routine procedures. Further investigation is needed to determine whether or not these new non-invasive methods have prognostic value or affect therapeutic choices in small cell lung carcinoma.

Bone marrow (BM) involvement is common in patients with small cell lung cancer (SCLC) although it is still not clear whether it is of prognostic value in this disease. In one study (Ihde *et al.*, 1981), BM metastases had little prognostic value, since the difference in survival rates (8 months versus 10 for patients with no BM involvement detected) was not statistically significant. In other series, BM involvement predicted a significantly lower median duration of response and median survival time (Hirsch & Hansen, 1980).

From a therapeutic point of view, BM involvement is known to increase haematological toxicity of conventional treatment (Hirsch & Hansen, 1980). Furthermore, undetectable BM tumour cells may be the cause of some of the relapses observed after intensive chemotherapy followed by autologous BM transplantation in SCLC (Spitzer *et al.*, 1986; Humblet *et al.*, 1987). Thus, knowledge of BM involvement at the time of diagnosis in SCLC is of great importance.

Routine detection of BM metastases in SCLC usually consists of unilateral posterior iliac crest aspiration and biopsy. With this procedure, BM involvement is found in 15–30% of patients (Hirsch & Hansen, 1980; Ihde & Hansen, 1981; Hansen *et al.*, 1978; Hirsch *et al.*, 1977; Anner & Drewinko, 1977; Choi & Carey, 1976; Holoye *et al.*, 1977). However, BM infiltration is detected in 35–66% of patients at necropsy (Kristjansen *et al.*, 1986; Ihde *et al.*, 1979). Furthermore, among SCLC patients with negative BM biopsy, BM involvement is seen in 8–11% with the use of *in vitro* semi-solid culture techniques (Carney *et al.*, 1980; Pollard *et al.*, 1981; Neumann *et al.*, 1984; Canon *et al.*, 1988), 22% with discontinuous gradient sedimentation method (Hunter *et al.*, 1987) and 50% with BM aspirates immunostained with monoclonal antibodies (MoAB) (Stahel *et al.*, 1985). Thus, routine cytological and histological examination of the BM is likely to underestimate greatly the rate of BM metastases in SCLC.

Magnetic resonance imaging (MRI) has recently been shown to be a very sensitive test for the detection of BM involvement in patients with lymphoma (Shields *et al.*, 1987) and neuroblastoma (Couanet & Jeoffray, 1988). We investigated the use of both MRI and immunological analysis with MoAB for BM staging in patients diagnosed with SCLC, and compared the results of these two new methods with conventional cytohistological methods.

Correspondence: V. Trillet.
Received 15 November 1988, and accepted in revised form 7 February 1989.

### Patients and methods

### *Patients*

From 15 April 1987 to 15 August 1988, 35 previously untreated patients with histologically proven SCLC were referred to our institution. There were 33 men and 2 women, with a median age of 61 years. Initial staging included routine blood tests, fibreoptic bronchoscopy + biopsies, CT scan of the thorax, abdominal ultrasonography and/or CT scan, brain CT scan and radionuclide bone scan (RBS) + BM staging (see below). With all these procedures, 27 out of 35 (77%) patients were considered to have metastatic disease at the time of diagnosis.

### *Cytological and histological examination of the BM*

In all patients, unilateral posterior iliac crest aspiration and biopsy were performed before treatment. Several smears were made from the aspirate and cytologic examination was done after Wright coloration.

The biopsies were performed according to the method described by Jamshidi & Swaim (1971) with a median length of 2.5 cm and touch imprints made from each biopsy. After decalcification, pathological slices were prepared with Haematoxylin and Eosin staining before light microscopic examination.

### *MoAB immunostaining*

In 33 of 35 patients, 2–3 ml of aspirated marrow were mixed with heparin and mononuclear BM cells were immediately obtained by Ficoll separation for immunological analysis. Two MoABs of the immunoglobulin (Ig) G isotype (UJ 13 A kindly provided by J. Kemshead, 11.14 kindly provided by J.C. Laurent, SANOFI) recognise antigens expressed by cells of neuroectodermal origin (Favrot *et al.*, 1986; Rosier &

Laurent, 1987), and CD45 of the IgM isotype (kindly provided by G. Janossy) recognises the panleucocyte antigen (Kemshead *et al.*, 1983; Maritaz *et al.*, 1988).

*Double immunofluorescence immunostaining* Two samples ($1.3 \times 10^6$ cells in suspension in 100 $\mu$l phosphate buffer saline (PBS) with 0.1% $NaN_3$) were incubated with the two MoABs (one for each sample) in combination with CD45. After 10 min at 24°C, samples were washed once in PBS and incubated with TRITC anti-mouse IgM specific and FITC anti-mouse IgG specific (Southern Biotechnology Associates) for 10 min at 24°C. Samples were then washed twice, maintained in PBS-glycerol and analysed in a fluorescent Zeiss microscope with a 40:1 objective, a 490 nm excitation filter and a K 530 barrier filter. Cells are considered malignant when positive with at least one of the 2 anti-neuroectodermal antigens MoABs and negative with the CD45.

*Alkaline phosphatase immunostaining* BM cells in suspension at $6 \times 10^5$ $ml^{-1}$ in PBS are cytocentrifuged into glass (100 $\mu$l per smear at 70 *g* for 5 min in a Shandon cytospin). Immunochemical staining is performed using an indirect 3-stage immunoenzymatic procedure with alkaline phosphatase (Dakopatts, Copenhagen, Denmark). Briefly, six air-dried slides are fixed for 3 min with acetone at 4°C, incubated for 60 min with MoABs (3 for UJ 13 A and 3 for 11.14) then for 30 min with enzyme-conjugated rabbit anti-mouse Ig (Dakopatts) and for 30 min with enzyme-conjugated swine anti-rabbit Ig (Dakopatts). Washes are done with Tris buffer. The final step consists of a 15 min incubation with Naphtol-As-Mx phosphate, dimethylformamide, levamisole and fast red (Sigma Co., St Louis, USA). Slides are counterstained with Haematoxylin, mounted permanently with glycerin and evaluated under an optical microscope. Negative controls without MoAB and positive controls with MoABs recognising class I antigen on normal BM cells are included in each test.

The limit of detection of those two immunological methods is of one malignant cell in $10^5$ normal mononuclear BM cells if $3 \times 10^6$ cells are analysed in double immunofluorescence and six smears in alkaline phosphatase (Maritaz *et al.*, 1988; Combaret *et al.*, 1989).

*Magnetic resonance imaging*

All patients were studied with a 0.5 T superconductive magnet (Magni Scan 5000 Thomson CGR) using a body coil and a large field of view (500 mm). Ten millimetre thick contiguous slices of the pelvic bony structures including femoral heads, upper extremities of the femurs, pelvic bones (iliac, sacrum), and two or three lower lumbar vertebral bodies were obtained in the coronal plane in order to include from anterior to posterior all the BM of these structures.

A T1-weighted spin echo sequence was performed using a TR of 500 ms and a TE of 26 ms. With this sequence, 16 contiguous slices are obtained within 10 min of positioning the patient within the coil. With this sequence, BM in normal adults is seen as homogeneous areas of high signal intensity. MR images were analysed by one operator.

MR examinations were considered to be positive or negative depending on the presence or absence of focal areas of low signal intensity within an area of normal signal intensity corresponding to marrow fat, as already described in malignant lymphoma (Shields *et al.*, 1987) and in SCLC (Trillet *et al.*, 1988).

*Radionuclide bone scan (RBS)*

RBS was performed in 32 out of the 35 patients, 2 h after a bolus intravenous injection of 9 MBq per kg hydroxymethyldiphosphonate labelled with $^{99}Tc^{m}$. Investigators were asked to provide an unambiguous result (positive or negative) and were blinded to the findings of the four other methods.

### Results

In eight patients, cytological examination of the BM was positive (Table I). BM biopsy was positive in only five out of these eight patients. In one case (no. 23), BM biopsy was positive with negative cytology. Thus, BM involvement was detected with cytological and/or histological examination in 9/35 (26%) cases: MRI was positive in all nine cases, BM involvement appearing as focal areas of low signal intensity within the normal signal intensity of marrow fat (Figures 1a and 2); this correlated with strongly positive immunostaining in all eight cases where it was performed with the percentage of malignant cells ranging from $10^{-4}$ to 0.6.

Of the 26 patients with negative conventional cytohistology, MRI and/or MoABs detected BM metastases in 10 (38%) cases: three had both positive MRI and immunostaining, six had positive MRI only, and one had positive immunostaining alone. MRI and immunostaining were negative in 16 of the 26 patients and in one case (no. 10) where immunostaining was not done, MRI was negative.

Five (14%) patients had extensive disease but no evidence of BM involvement using conventional and specialised techniques.

MoABs UJ 13 A and 11-14 stained more than 95% SCLC tumour samples with very homogeneous staining of malignant cells. Table II shows the concordance between immunofluorescence and alkaline phosphatase immunostaining. A limitation to the use of MoABs against SCLC on BM samples is their reactivity with very few normal cells of the NK lineage. The use of double immunofluorescence analysis permits one to accurately distinguish between those few normal cells (stained with UJ 13A, 11-14 and the anti-panleucocyte MoAB) and malignant cells negative with the anti-

**Table I** Results of MoAB immunostaining and MRI findings compared to routine procedures

| *Stage* | *Patient* | *Cytology* | *BM biopsy* | *MoAB* | *MRI* | *RBS* |
|---|---|---|---|---|---|---|
| ED | 1 | + | + | ND | + | + |
| ED | 2 | + | + | + | + | − |
| ED | 22 | + | + | + | + | + |
| ED | 26 | + | + | + | + | + |
| ED | 29 | + | + | + | + | + |
| ED | 3 | + | − | + | + | − |
| ED | 24 | + | − | + | + | − |
| ED | 15 | + | − | + | + | ND |
| ED | 23 | − | + | + | + | + |
| LD | 25 | − | − | + | + | + |
| ED | 11 | − | − | + | + | ND |
| ED | 31 | − | − | + | + | + |
| ED | 12 | − | − | − | + | + |
| ED | 17 | − | − | − | + | + |
| ED | 16 | − | − | − | + | − |
| ED | 35 | − | − | − | + | − |
| ED | 20 | − | ND | − | + | + |
| ED | 32 | − | ND | − | + | + |
| ED | 14 | − | − | + | − | − |
| ED | 4 | − | − | − | − | − |
| ED | 5 | − | − | − | − | − |
| ED | 6 | − | − | − | − | − |
| ED | 7 | − | − | − | − | − |
| ED | 8 | − | − | − | − | − |
| LD | 18 | − | − | − | − | − |
| LD | 21 | − | − | − | − | − |
| LD | 28 | − | − | − | − | − |
| LD | 9 | − | − | − | − | ND |
| LD | 30 | − | − | − | − | − |
| LD | 33 | − | − | − | − | − |
| LD | 34 | − | − | − | − | − |
| ED | 13 | − | − | − | − | +* |
| ED | 19 | − | − | − | − | +* |
| ED | 27 | − | − | − | − | +* |
| LD | 10 | − | − | ND | − | − |

ND, not done; +, positive; −, negative; LD, limited disease; ED, extensive disease; *, RBS was positive in areas where MRI was not performed.

**Table II** Results of MoAB immunostaining

| *Patient* | *IF* | *AP* | *Percentage of malignant cells quantified by the immunostaining* |
|---|---|---|---|
| 2 | + | + | $10^{-4}$ |
| 22 | ND | + | $6\times10^{-1}$ |
| 26 | + | + | $2\times10^{-1}$ |
| 29 | + | + | $2\times10^{-1}$ |
| 3 | + | ND | $10^{-1}$ |
| 24 | + | + | $10^{-2}$ |
| 15 | + | + | $10^{-3}$ |
| 23 | + | ND | $10^{-2}$ |
| 25 | ND | + | $10^{-5}$ |
| 11 | − | + | $10^{-5}$ |
| 31 | + | ND | $10^{-3}$ |
| 14 | + | ND | $10^{-3}$ |

ND, not done; +, positive; −, negative; IF, double immunofluorescence; AP, alkaline phosphatase.

panleucocyte MoAB. The morphological control used with the alkaline phosphatase method also provides objective criteria for malignancy.

RBS (Table I) provided evidence of cortical bone involvement in 14 cases, 11 of which also had positive MRI. The remaining patients (nos. 13, 19 and 27) with negative MRI were shown to have bone metastases of the sternum or the dorsal spinal column, locations not studied by MRI. Five patients had positive MRI and normal RBS. Bone CT scan was performed in two of them and no abnormality was seen at the location of the MRI abnormal areas (Figure 1b).

In one (no. 2) of the 27 patients with extensive disease, BM was the only metastatic site and BM invasion was detectable by cytohistological examination as well as by MoAB immunostaining and MRI. Nine out of the 10 patients with positive MRI and/or MoAB examination of the BM and a negative routine examination were shown to have at least one other metastatic site at diagnosis. Thus, only one patient (no. 25) was shifted from the 'limited disease' group to the 'extensive disease' group by including these two new methods in the initial staging.

### Discussion

In our series, it appears that analysis of one BM aspirate was positive in three cases where biopsy was negative, whereas there was only one example of false negative cytology compared to histological examination of the BM. These results are consistent with the conclusions of Hirsch and co-workers (Hirsch *et al.*, 1977) on a group of 203 SCLC patients where BM aspiration was shown to be more effective than BM biopsy in detecting BM metastases in SCLC. On the contrary, Lawrence (Lawrence *et al.*, 1984) claimed that histological examination of the BM was superior, when long enough BM specimens were obtained, that is, with the use of the Jamshidi needle instead of the shorter Radner needle used by the Danish group. But in most instances, his patients underwent a second and even a third biopsy 'through the same skin incision from a location of a few millimeters lateral to the first'.

This is in accordance with previous reports on many neoplastic diseases (Brunning *et al.*, 1975) including SCLC (Hirsch *et al.*, 1979) which demonstrate that the number of positive BM aspirates and biopsies increases with bilateral examination of the BM. The rate of detection of BM involvement is clearly related to the total amount of BM tissue analysed and this is thought to result from the focal pattern of BM invasion by malignant cells (Lawrence *et al.*, 1984). However, invasive procedures such as bilateral iliac crest trephines are often considered aggressive and investigators have been encouraged to develop new non-aggressive methods of detection.

Immunological analysis is a highly sensitive method and allows detection of malignant cells in roughly 50% SCLC patients using MoABs directed against SCLC cells surface antigens like SM1 (Stahel *et al.*, 1985) or MOC (Postmus, personal communication). Here, the concentration of malignant cells by Ficoll separation, the analysis of a very large number of cells and the use of objective criteria of malignancy such as morphologic control (alkaline phosphatase method) and double membrane immunostaining with two different markers (double immunofluorescence method) improves the level of detection to $10^{-5}$ cells and allows the identification of malignant cells with cytological lymphoid-like features. MoAB immunostaining allows an estimate of the proportion of malignant cells. In the four cases where MoAB immunostaining was positive while routine procedures were negative, the contamination in the aspirate was low (less than or equal to $10^{-3}$). In one of our patients, MoAB immunostaining was the only positive test.

Of the nine patients with positive MRI and negative cytology and biopsy, three showed positive immunostaining of the BM and, in the remaining six patients, the signals obtained with MRI were very similar to those observed in cytologically positive BM patients, and similar to those described as typical features of BM involvement leukaemias, lymphomas, neuroblastomas and SCLC. Furthermore, patterns of positivity (multiple focal areas of hyposignals) tend to confirm the focal nature of BM involvement and may explain the insensitivity of methods based on the examination of a single aspiration or biopsy site, including immunological analysis. MRI has the advantage of being painless, being able to scan a wide part of the body and being particularly sensitive to focal involvement. There is a need for the confirmation of malignant BM invasion in the sites of MRI hyposignals, in patients with normal BM aspiration and normal biopsy in one iliac crest.

RBS is a highly sensitive but very non-specific test in SCLC (Levenson *et al.*, 1981). In our study, all RBS-positive patients also had a positive MRI, except for three cases where bone metastases were detected outside the MRI field of view. In contrast, in five patients with positive MRI, RBS was negative. MRI focal areas of low intensity are located in the fat tissue, that is, in BM itself (Porter *et al.*, 1986). In contrast, RBS abnormalities reflect cortical bone involvement. Therefore, no comparison can be made between these two methods in terms of sensitivity and specificity. However, the lack of bone metastases detected with RBS and CT bone scan in MRI-positive areas might reflect different patterns of BM and cortical bone involvement in SCLC. According to our results with MRI, metastatic spread in the BM could be an earlier event than cortical bone involvement with SCLC. This sequence of bone invasion is concordant with the figures shown in our study: the rate of BM metastases detected with MRI (50%) is greater than the usual rate of bone metastases (30–35% in most series) (Ihde & Hansen, 1981).

The necessity of BM sampling in SCLC has recently been questioned by some authors (Campling *et al.*, 1986). According to our preliminary results, routine cyto-histological examination of the BM might fail to detect BM involvement in 38% of patients with SCLC and BM could be a very frequent site of detectable malignant cells in SCLC using MRI and MoAB immunostaining. In our opinion, in a disease where the presence and number of metastatic sites is known to be an important prognostic factor, this finding strongly supports routine examination of the BM with these two complementary and non-aggressive methods. Furthermore, if future results confirm the superiority of cytological and immunological examination of BM aspiration compared to biopsy, the latter aggressive method could possibly be abandoned.

The fact that some patients might be diagnosed as having metastatic instead of limited disease could be of a prognostic importance since it could improve the selection of truly 'limited stage' patients to be treated with a 'curative intent'.

However, compared to the series of Carney *et al.* (1988) where 45% of the patients were restaged after MRI examination of the BM, we found only one patient with apparently 'limited disease' where MRI and/or MoAB immunostaining resulted in the re-classification in the 'extensive disease' category. This is possibly due both to different patient selection (since 23% of our patients were in the 'limited disease' group versus 60% in Carney's study) and to better routine evaluation of the BM (26% cytologically and/or histologically positive BM instead of 5%).

Another major clinical application for MRI and immunostaining could be the follow-up of BM metastases shown by MRI after chemotherapy to better evaluate the response to therapy which is known to be a major prognostic factor in this disease.

This report is part of an ongoing study undertaken by our institution in order to determine prospectively in a larger series of patients the prognostic and therapeutic implications of the use of these two complementary and non-aggressive methods of tumour staging.

We wish to thank Dr T. Philip (Centre Léon Bérard, Lyon, France) for his help and advice in the initiation of this work, Drs J. Armitage (University of Omaha, Nebraska), S. Narod and G.M. Lenoir (International Agency for Research on Cancer, Lyon, France) for review of the manuscript.

**Figure 1** (**a**) Coronal T1-weighted MRI of patient no. 2 with BM involvement. Notice the well-circumscribed areas of low signal intensity within the sacrum. (**b**) In the same patient, CT scan does not demonstrate any evidence of bone abnormalities.

**Figure 2** In this patient (no. 3), T1-weighted spin-echo image demonstrates major BM involvement: all lumbar vertebral bodies and the upper extremities of both femoral heads present diffuse areas of heterogeneous low intensity signals.

### References

ANNER, R.M. & DREWINKO, B. (1977). Frequency and significance of bone marrow involvement by metastatic solid tumors. *Cancer*, **39**, 1337.

BRUNNING, R.D., BLOOMFIELD, C.D., McKENNA, R.W. and 1 other (1975). Bilateral trephine bone marrow biopsies in lymphoma and other neoplastic diseases. *Ann. Intern. Med.*, **82**, 365.

CAMPLING, B., QUIRT, I., DEBOER, G. and 3 others (1986). Is bone marrow examination in small cell lung cancer really necessary? *Ann. Intern. Med.*, **105**, 508.

CANON, J.L., HUMBLET, Y. & LEBACQ-VERHEYDEN, A.M. (1988). Immunodetection of small cell lung cancer metastases in bone marrow using three monoclonal antibodies. *Eur. J. Cancer Clin. Oncol.*, **24**, 147.

CARNEY, D.N., GAZDAR, A.F. & MINNA, J.D. (1980). Positive correlation between histological tumor involvement and generation of tumor cells colonies in agarose in specimen taken directly from patients with small-cell carcinoma of the lung. *Cancer Res.*, **40**, 1820.

CARNEY, D., REDMOND, O., HARFORD, P. and 2 others (1988). Bone marrow involvement by small cell lung cancer using magnetic resonance imaging. *Lung Cancer*, **4** (suppl.), A 45.

CHOI, C.H. & CAREY, R.W. (1976). Small cell anaplastic carcinoma of lung. *Cancer*, **37**, 2651.

COMBARET, V., FAVROT, M.C., KREMENS, B. and 6 others (1989). Immunological detection of neuroblastoma cells in the bone marrow harvested for autologous transplantation. *Br. J. Cancer* (in the press).

COUANET, D. & JEOFFRAY, A. (1988). Etude en imagerie par résonnance magnétique (IRM) des métastases ostéo-médullaires des neuroblastomes. *Bull. Cancer*, **75**, 91.

FAVROT, M.C., FRAPPAZ, D., MARITAZ, O. and 8 others (1986). Histological, cytological and immunological analyses are complementary for the detection of neuroblastoma cells in bone marrow. *Br. J. Cancer*, **54**, 637.

HANSEN, H.H., DOMBERNOWSKY, P. & HIRSCH, F.R. (1978). Staging procedures and prognostic features in small anaplastic bronchogenic carcinoma. *Semin. Oncol.*, **5**, 280.

HIRSCH, F., HANSEN, H.H., DOMBERNOWSKY, P. and 1 other (1977). Bone marrow examination in the staging of small cell anaplastic carcinoma of the lung with special reference to subtyping: an evaluation of 203 consecutive patients. *Cancer*, **39**, 2563.

HIRSCH, F.R., HANSEN, H.H. & HAINAU, B. (1979). Bilateral bone marrow examinations in small cell anaplastic carcinoma of the lung. *Acta Pathol. Microbiol. Scand.*, **87**, 59.

HIRSCH, F. & HANSEN, H.H. (1980). Bone marrow involvement in small cell carcinoma of the lung: prognostic and therapeutic aspects. *Cancer*, **46**, 206.

HOLOYE, P.Y., SAMUELS, M.L. & LANZOTTI, V.I. (1977). Combination chemotherapy and radiation therapy for small cell carcinoma. *JAMA*, **237**, 1221.

HUMBLET, Y., SYMANN, M., BOSLY, A. and 8 others (1987). Late intensification chemotherapy with autologous bone marrow transplantation in selected small cell carcinoma of the lung: a randomized study. *J. Clin. Oncol.*, **5**, 1864.

HUNTER, R.F., BRODWAY, P., SUN, S. and 2 others (1987). Detection of small cell lung cancer bone marrow involvement by discontinuous gradient sedimentation. *Cancer Res.*, **47**, 2737.

IHDE, D.C., SIMMS, E.G., MATTHEWS, M. and 3 others (1979). Bone marrow metastasis in small cell carcinoma of the lung: frequency, description, and influence on chemotherapeutic toxicity and prognosis. *Blood*, **53**, 677.

IHDE, D.C. & HANSEN, H.H. (1981). Staging procedures in small cell carcinoma of the lung. In *Small Cell Lung Cancer*, Greco, I.A., Oldhan, R.K. & Bunn, P.A. (eds) p. 261. Grune and Stratton: New York.

IHDE, D.C., MAKUCH, R.W., CARNEY, D.N. and 3 others (1981). Prognostic implications of stage of disease and sites of metastases in patients with small cell carcinoma of the lung treated with intensive combination chemotherapy. *Am. Rev. Respir. Dis.*, **123**, 500.

JAMSHIDI, K. & SWAIM, W.R. (1971). Bone marrow biopsy with unaltered architecture. A new biopsy device. *J. Lab. Clin. Med.*, **77**, 335.

KEMSHEAD, J.T., GOLDMAN, A., FRITSCHY, J. and 2 others (1983). Use of panels of monoclonal antibodies in the differential diagnosis of neuroblastoma and lymphocytic disorders. *Lancet*, **i**, 12.

KRISTJANSEN, P.E.G., OSTERLIND, K. & HANSEN, M. (1986). Detection of bone marrow relapse in patients with small cell carcinoma of the lung. *Cancer*, **58**, 2538.

LAWRENCE, J.B., ELEFF, M., BEHM, F.G. and 1 other (1984). Bone marrow examination in small cell carcinoma of the lung: comparison of trephine biopsv with aspiration. *Cancer*, **53**, 2188.

LEVENSON, R.M., SAUERBRUNN, B.J.L. & IHDE, D.C. (1981). Small cell lung cancer: radionucleide bone scans for assessment of tumor extent and response. *AJR*, **137**, 31.

MARITAZ, O., COMBARET, V. & FAVROT, M.C. (1988). Intérêt de l'analyse immunologique pour la détection de neuroblastes résiduels dans la moëlle osseuse. *Path. Biol.*, **36**, 21.

NEUMANN, H.A., LOHR, G.N. & FAUSER, A.A. (1984). Tumor cell colonies in bone marrow cultures from patients with small cell carcinoma of the lung. *Blut*, **48**, 227.

POLLARD, E.B., TIO, F., MYERS, J.W. and 3 others (1981). Utilization of a human tumor cloning system to monitor for bone marrow involvement with small cell carcinoma of the lung. *Cancer Res.*, **41**, 1015.

PORTER, B.A., SHIELDS, A.F. & OLSON, D.O. (1986). Magnetic resonance imaging of bone marrow disorders. *Radiol. Clin. North Am.*, **24**, 269.

ROSIER, F. & LAURENT, J.C. (1987). Analysis of related antigens of a group of antibodies having small cell lung cancer cell and neural reactivity using reciprocal binding inhibition experiments. In *Proceedings of the First International Workshop on SCLC Markers*, London.

SHIELDS, A.F., PORTER, B.A., OLSON, D.O. and 2 others (1987). The detection of bone marrow involvement by lymphoma using magnetic resonance imaging. *J. Clin. Oncol.*, **5**, 225.

SPITZER, G., FARHA, P., VALDIVIESO, M. and 8 others (1986). High dose intensification therapy with autologous bone marrow support for limited small-cell bronchogenic carcinoma. *J. Clin. Oncol.*, **4**, 4.

STAHEL, R.A., MABRY, M., SKARIN, A.T. and 2 others (1985). Detection of bone marrow metastasis in small cell lung cancer by monoclonal antibody. *J. Clin. Oncol.*, **3**, 455.

TRILLET, V., REVEL, D., LOIRE, R. and 7 others (1988). The detection of bone marrow involvement in small cell lung cancer using magnetic resonance imaging. *J. Clin. Oncol.*, **6**, 397.